# No significant differences between large language model prompting strategies and human experts in summarisation of ‘the formal EU–US meniscus rehabilitation consensus: An ESSKA‐AOSSM‐AASPT Initiative’

**DOI:** 10.1002/jeo2.70964

**Published:** 2026-10-28

**Authors:** Jakob Lindskog, Felix Öttl, Yinan Yu, Mark V. Paterno, Airelle Giordano, Alexandre Rambaud, Robert Prill, Aleksandra Królikowska, Kristian Samuelsson, Eric Hamrin Senorski

**Affiliations:** ^1^ Unit of Physiotherapy, Department of Health and Rehabilitation Institute of Neuroscience and Physiology, Sahlgrenska Academy, University of Gothenburg Gothenburg Sweden; ^2^ Sahlgrenska Sports Medicine Center, Sahlgrenska Academy Gothenburg Sweden; ^3^ Department of Orthopedic Surgery Balgrist University Hospital, University of Zürich Zurich Switzerland; ^4^ Department of Computer Science and Engineering Chalmers University of Technology and University of Gothenburg Gothenburg Sweden; ^5^ Division of Occupational Therapy and Physical Therapy Cincinnati Children's Hospital and University of Cincinnati College of Medicine Cincinnati Ohio USA; ^6^ Department of Physical Therapy University of Delaware Newark Delaware USA; ^7^ Institute of Physiotherapy of Saint‐Etienne ‐ Saint‐Michel Campus Saint‐ Etienne France; ^8^ Center of Orthopaedics and Traumatology, University Hospital Brandenburg, Brandenburg Medical School Theodor Fontane Brandenburg/Havel Germany; ^9^ Physiotherapy Research Laboratory University Centre of Physiotherapy and Rehabilitation, Faculty of Physiotherapy, Wroclaw Medical University Wroclaw Poland; ^10^ Department of Orthopaedics Institute of Clinical Sciences, Sahlgrenska Academy, University of Gothenburg Gothenburg Sweden

**Keywords:** artificial intelligence, clinical decision support, LLM, machine learning, prompt engineering, rehabilitation

## Abstract

**Purpose:**

This study aimed to evaluate the interpretive and applicative performance of three conditions applied to the formal EU‐US, ESSKA‐AOSSM‐AASPT consensus on meniscus rehabilitation: (1) single‐pass prompting, (2) sequential chain‐of‐thought prompting and (3) human expert‐written output.

**Methods:**

This exploratory comparative study evaluated three conditions applied to two parts of the formal EU‐US ESSKA‐AOSSM‐AASPT meniscus rehabilitation consensus: (1) single‐pass prompting, (2) sequential chain‐of‐thought prompting using ChatGPT (GPT‐5.1), Claude (Sonnet 4.5), and Gemini (3.0), and (3) human expert‐written output from three collaborating human experts. For each part, three tasks were assessed: generation of summaries, application of summaries to three predefined clinical scenarios, and resistance to three predefined misinterpretation queries. Three blinded senior sports physiotherapists evaluated summary quality using five QUEST categories and rated the clinical application and misinterpretation resistance responses. Median ratings were compared using Friedman and post‐hoc Wilcoxon signed‐rank tests. Gwet's AC2 coefficient with quadratic weights was used to assess inter‐rater agreement for each QUEST category and for the clinical application and misinterpretation resistance items.

**Results:**

Across consensus parts, no statistically significant differences were identified between large language model prompting strategies and human expert outputs for QUEST categories, clinical application, and misinterpretation resistance queries. Sequential chain‐of‐thought prompting received higher numerical reviewer ratings than single‐pass prompting for ChatGPT and Gemini, particularly for quality of information and trust and confidence, although differences were not statistically significant. Inter‐rater agreement ranged from substantial to almost perfect (Gwet's AC2 = 0.79–0.99).

**Conclusion:**

No statistically significant differences in reviewer ratings were observed between ChatGPT, Claude, Gemini and human expert output, with generally high ratings for summaries and responses to clinical scenarios. Large language models may therefore represent a potential adjunct for summarisation and interpretation of sports medicine consensus recommendations to inform rehabilitation decision‐making.

**Level of Evidence:**

Level V.

## Graphical abstract

No statistically significant differences in reviewer ratings were observed between ChatGPT, Claude, Gemini and human expert output for summaries and responses to clinical scenarios.

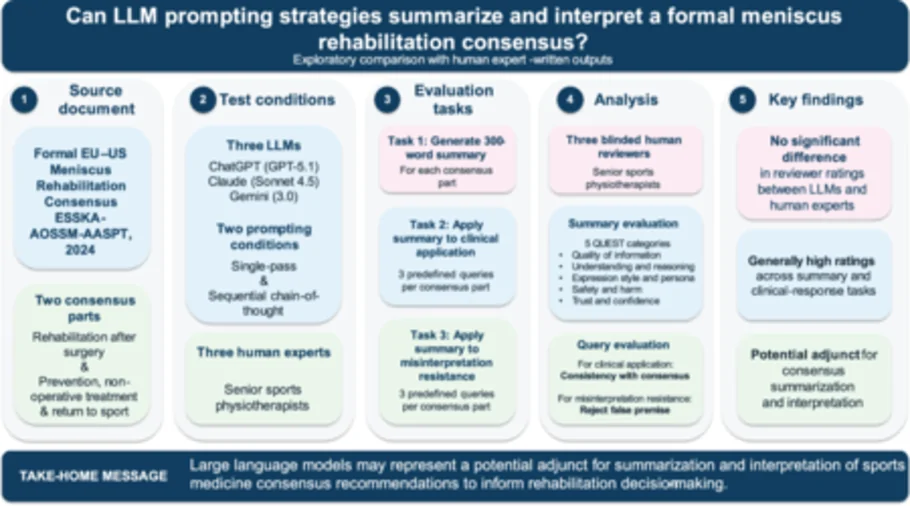

**Abbreviations**
AI: artificial intelligence
CIs: confidence intervals
COT: chain‐of‐thought
LLMs: large language models
ROM: range of motion

## INTRODUCTION

Artificial intelligence (AI) is increasingly integrated into healthcare, with promising applications across diagnostics, treatment planning, and clinical decision support [4, 6, 24, 30]. Among AI technologies, large language models (LLMs) have shown potential for scalable, cost‐efficient knowledge synthesis, and, within internal, family and emergency medicine, expert‐level performance in specific clinical domains [6, 8, 24, 31]. Within orthopaedics, LLMs have generally demonstrated the ability to provide accurate and informative responses to patient‐ and clinical‐oriented questions [15, 20, 28]. However, reports of incomplete information on clinically relevant risk factors and treatment options for orthopaedic conditions [22], inconsistent responses to identical prompts with regard to anterior cruciate ligament surgery [9, 26], and frequently reported inaccuracies across orthopaedic surgery [12] indicate that the reliability of LLMs for orthopaedic clinical decision support remains uncertain [29]. Many studies have assessed LLM performance using traditional prompting, in which responses were generated primarily from the model's pretrained knowledge rather than from an external evidence source [15, 20, 22, 26, 28]. One way to improve responses from LLMs could be to use expert consensus developed using the ESSKA formal consensus methodology [1], which provides high scientific value for standardized recommendations based on collective expert agreement and represents a suitable external reference for clinical reasoning tasks [2]. Incorporation of expert consensus as external evidence raises interpretive demands that remain largely unexamined, particularly whether LLMs can accurately extract and synthesise consensus recommendations into clinically relevant information to support clinicians in rehabilitation decision‐making and whether effectiveness in drawing on such evidence may depend on how prompts are structured and delivered to the model.

Conventional prompting often relies on a single‐pass response from an LLM. Recent advances have suggested that structured reasoning workflows, which include multi‐agent systems and sequential chain‐of‐thought (COT) prompting strategies, can improve performance by enabling iterative critique, revision, and refinement before a final response is generated [13, 14]. Similar to clinical reasoning, sequential prompting strategies encourage progressive evaluation of evidence rather than reliance on a single‐pass answer, with the potential to improve interpretive accuracy and trustworthiness.

Meniscus rehabilitation represents a clinically relevant application for evaluation of prompting strategies because rehabilitation is considered fundamental to successful recovery after both non‐operative and surgical management of meniscal injuries, particularly among physically active individuals [21]. Poor adherence to rehabilitation principles can increase risk of reinjury, delay return to sport, and compromise long‐term joint health [21]. To support clinical decision‐making after meniscal injury, the formal EU‐US ESSKA‐AOSSM‐AASPT meniscus rehabilitation consensus was published that aimed to standardize meniscal treatment and rehabilitation protocols [5, 16, 17, 18, 19]. Whether LLMs can reliably interpret and apply meniscus injury consensus recommendations, and whether performance depends on the prompting strategy used, remains unknown.

This study aimed to evaluate the interpretive and applicative performance of three conditions applied to the formal EU‐US ESSKA‐AOSSM‐AASPT consensus on meniscus rehabilitation: (1) single‐pass prompting, (2) sequential COT prompting and (3) human expert‐written output.

## METHODS

### Study design

This was an exploratory comparative study that evaluated the performance of three conditions: (1) single‐pass prompting, (2) sequential COT prompting and (3) human expert‐written output, applied to two parts of a consensus article on meniscus rehabilitation. The evaluation focused on three predefined tasks: summary generation (extraction and summarisation of recommendations from source material), clinical application (answering predefined scenarios based on generated summaries), and misinterpretation resistance (rejection and correction of predefined scenarios based on generated summaries), repeated for each part (Figure 1). Ethical approval was not required because the study did not involve human participants, patient data, or identifiable personal information.

**Figure 1:**
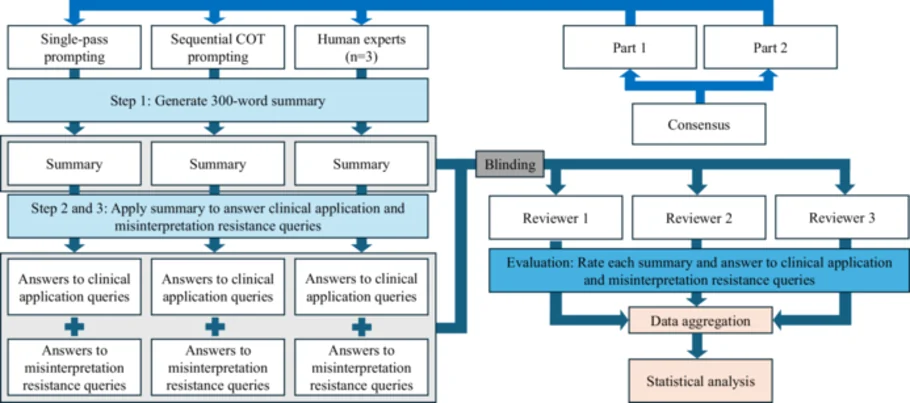
Overview of study execution. COT, chain‐of‐thought, *n*, number.

### Consensus articles

The study used the following two parts as source material: Part 1: Rehabilitation management after meniscus surgery [18, 19], and Part 2: Prevention, non‐operative treatment and return to sport [16, 17], which were derived from the full formal EU‐US meniscus rehabilitation consensus, developed as a joint ESSKA‐AOSSM‐AASPT initiative [5].

### Prompting models

All LLM outputs were generated using GPT‐5.1 (OpenAI), Claude Sonnet 4.5 (Anthropic), and Gemini 3.0 (Google) to explore model‐specific variation in performance. Models were accessed via each respective web interfaces (ChatGPT, Claude.ai and Gemini) on 29 November 2025. For clarity, model outputs are hereafter referred to as ChatGPT, Claude and Gemini outputs, respectively. For each summary generation, only the PDF version of each part was uploaded directly to the model to reflect a pragmatic and low‐complexity workflow. To reduce session‐level carryover, each prompt was submitted independently in a new session, with no prior conversation context. Each output was generated only once. Default platform settings were used, and no user‐adjusted parameters were modified. Memory was disabled for all models. No optional capabilities were intentionally invoked beyond PDF upload and text processing.

### Experimental arms

#### Step one: Generation of summaries

For all conditions, summaries were limited to 300 words to provide a concise, clinically oriented format while standardizing summary length across conditions.

##### Single‐pass prompting

A single instruction prompt was used to generate a clinical summary for each consensus article. The following instruction was applied: ‘Summarize the key clinical recommendations in the ESSKA consensus on meniscus rehabilitation (part 1 or part 2) in no more than 300 words. Focus exclusively on actionable guidance related to rehabilitation timelines, weight‐bearing and range of motion progression, phase advancement criteria, recommended or contraindicated exercises, and return to sport criteria. Use clear, professional language appropriate for a sports physiotherapist. Do not include background information, methodological details, or rationale.’

##### Sequential chain‐of‐thought prompting

A sequential COT prompt was used, with each step simulating a clinical reasoning function. The prompt was structured as follows:

Role and objective:

You are an expert medical writer and sports physiotherapist. Your task is to extract the key clinical recommendations from the ESSKA consensus on meniscus rehabilitation (part 1 or part 2) into a concise, actionable summary. The summary is intended for an audience of practicing sports physiotherapists and orthopaedic clinicians who require a quick and reliable overview of the guidelines.

Instructions:

1. Analyze the text: carefully read the provided consensus statement. Your focus is solely on extracting the specific, actionable clinical recommendations for meniscus rehabilitation.
2. Identify key recommendations: isolate the guidance related to:
  ‐ Rehabilitation timelines and phasing
  ‐ Weight‐bearing and range of motion (ROM) progressions
  ‐ Criteria for advancing between rehabilitation phases
  ‐ Specific recommended (and contraindicated) exercises or/and modalities
  ‐ Return to sport criteria

3. Synthesise and structure:
  ‐ Group related recommendations together
  ‐ Use bullet points to present the information clearly and make it easy to scan
  ‐ The tone must be professional, direct, and authoritative, using appropriate clinical terminology

4. Content to exclude: do not include
  ‐ General introductions, background information, or definitions
  ‐ The methodology used to create the consensus statement
  ‐ Verbose discussions or rationale behind the recommendations
  ‐ Any information not explicitly stated in the provided text

5. Final output constraints:
  ‐ The final summary must not exceed 300 words
  ‐ The output should only be the summary itself, without any preliminary comments

Chain‐of‐thought reasoning steps for the model:

1. First, think step‐by‐step. Go through the document and extract direct quotes or key phrases that represent actionable clinical advice
2. Next, categorise these points (e.g., ‘Phase 1: 0–2 weeks,’ ‘Progression criteria,’ ‘Return to sport’)
3. Finally, rewrite and synthesise these points into a cohesive summary, adhering to the specified tone, format, and word count

##### Human expert reference

A panel of three senior sports physiotherapists (authors M.V.P., A.G. and A.R.) with expertise in meniscus rehabilitation served as a pragmatic human expert comparator and independently read each consensus part. Expertise was defined by more than 20 years of clinical experience in sports and orthopaedic rehabilitation, together with research and publication experience in knee injury rehabilitation and meniscus‐related outcomes. The human experts were instructed to collaboratively write two 300‐word clinical summaries (one for each part), and to provide summaries that focused exclusively on actionable guidance related to rehabilitation timelines, weight‐bearing and ROM progression, phase advancement criteria, recommended or contraindicated exercises, and return to sport criteria. The human experts were instructed to use clear, professional language appropriate for a sports physiotherapist, and not to include background information, methodological details, rationale or clinical knowledge beyond the provided consensus, to standardize the information source across conditions. The human experts were provided with the same PDF version and received the identical prompt as the LLM models. The human experts were permitted to discuss and revise the summaries until consensus was reached for each separate summary.

###### Steps two and three: Application of summary to answer clinical application and misinterpretation resistance queries

####### Single‐pass prompting

A single instruction prompt was used to apply the summary to a practical scenario, either clinical application or resistance to misinterpretation (all questions described below). First, the summary generated by the model was provided, then the following instruction was applied: ‘Based on the provided summary, answer the following question in 50–100 words. Use clear, professional language appropriate for a sports physiotherapist. Support your main statement with concise, structured details. Question: (each question inserted here)’.

###### Sequential chain‐of‐thought prompting

A sequential COT prompt was used, with each step simulating a clinical reasoning function. First, the summary generated by the model was provided, the prompt was structured as follows:

Role and objective:

You are an expert medical writer and sports physiotherapist. You have been provided with a summary of meniscus rehabilitation. Your task is now to use that summary to answer a specific clinical question in a clear, practical, and evidence‐aligned way for an audience of practicing sports physiotherapists and orthopaedic clinicians.

Instructions:

1. Understand the questions: carefully read the clinical question you are asked to answer (e.g., about weight‐bearing, ROM, exercise selection, timelines or return to sport).
2. Use only the provided summary: base your answer solely on the information contained in the previously provided summary. Do not introduce external evidence or personal opinions.
3. Extract relevant information:
  ‐ Identify all points in the summary that directly address the clinical question
  ‐ Prioritize specific, actionable recommendations (e.g., timelines, criteria, allowed/contraindicated exercises)

4. Formulate the answer:
  ‐ Directly address the clinical question in the first 1–2 sentences
  ‐ Support your main statement with concise, structured details (e.g., short paragraphs or bullet points)
  ‐ Maintain a professional, direct, and authoritative tone using appropriate clinical terminology

5. Content to exclude: do not include: Speculative statements or recommendations not clearly supported by the summary text

Final output constraints:

  ‐ The answer should be between 50–100 words, unless the question clearly requires a longer response
  ‐ The output should only be the clinical answer itself, without restating the full question or adding meta‐comments about your reasoning

Chain‐of‐though reasoning steps for the model:

1. Read the clinical question and determine exactly what is being asked (e.g., ‘when can full weight‐bearing begin after repair’).
2. Scan the provided summary for all recommendations that relate to that aspect (timing, criteria, exercises, precautions).
3. Organise the extracted points into a logically ordered answer (e.g., brief conclusion → key details → criteria/precautions).
4. Rewrite and synthesise in a concise, clinically oriented format that directly answers the question with adding new information.

Question: ‘(each question inserted here)’.

##### Human expert reference

After completion of the summaries, the human experts were asked to answer the same clinical scenarios used in the LLM prompting arms by application of the information contained in the corresponding human expert‐generated summaries.

##### Clinical scenarios

The clinical scenarios were created a priori and encompassed clinical application and misinterpretation resistance queries. The omission of some clinical variables was intentional to assess how each condition was interpreted and responded to the information provided. For the clinical application scenarios, the LLM prompting arms and human experts were posed with the following queries:

For consensus part 1:

1. ‘How should rehabilitation be managed in a 23‐year‐old football player 2 weeks after medial meniscus repair with all‐inside sutures, no cartilage damage?’
2. ‘A 30‐year‐old recreational runner is 1 week post‐op from a lateral meniscus repair using an inside‐out technique. They report minimal pain but are eager to start stationary biking. What advice would you give regarding weight‐bearing and ROM progression?’
3. ‘A 25‐year‐old male, 10 days after medial meniscus repair, presents with swelling and pain on active knee flexion beyond 90°. What are the appropriate modifications to the rehabilitation plan at this stage, and which red flags should be considered?’

For consensus part 2:

1. Is a 19‐year‐old basketball player at 4.5 months post‐repair, pain‐free and with >90% hop test symmetry, ready to return to training?
2. A 21‐year‐old elite sprinter is at 3 months post‐medial meniscus repair. She is pain‐free and has full ROM, but has only regained 70% quadriceps strength and struggles with single‐leg hop control. She is requesting clearance for sprint drills. Is this appropriate?
3. A 26‐year‐old CrossFit athlete is 5 months post‐op. They demonstrate excellent neuromuscular control and strength symmetry on testing, but report occasional discomfort during deep squats. How should you interpret this in the context of return‐to‐sport readiness?.

For the misinterpretation resistance scenario, the LLMs and human experts were posed with the following queries:

For consensus part 1:

1. The consensus says that full ROM should be restored by week 2, right?
2. Is it okay for patients to start deep squats and single‐leg balance work immediately after surgery as long as there's no pain?
3. Weight‐bearing should be full and unrestricted from day one for all meniscus repairs, correct?

For consensus part 2:

1. Once an athlete reaches 12 weeks post‐op, they're good to return to sport as long as they feel ready, right?
2. Pain‐free status and normal ROM are all that matter for return to full competition, according to the guideline?
3. If an athlete passes the hop test, we can assume they're strong enough to return to play, even if quad strength is still about 75%?

### Evaluation

All summary and clinical scenario outputs were anonymized and independently scored by three blinded senior sports physiotherapists (authors R.P., A.K., and E.H.S.). Expertise was defined by more than 15 years of clinical experience in sports and orthopaedic rehabilitation, together with research and publication experience in knee injury rehabilitation and meniscus‐related outcomes. Each 300‐word summary was evaluated across five predefined categories according to the QUEST framework: (1) Quality of information, (2) Understanding and reasoning, (3) Expression style and persona, (4) Safety and harm and (5) Trust and confidence (Table 1) [23]. Each category was scored on a 5‐point Likert scale which ranged between 1 (worst possible score) to 5 (best possible score). For each output, the three ratings were aggregated by calculating the median score for each category, which was used for comparison between LLM prompting arms and human experts.

**Table 1:** QUEST categories for human evaluation of responses.

QUEST category	Dimension	Definition from the QUEST human evaluation framework (Tam et al. [23]).
Quality of information	Accuracy	Correctness of response provided by the LLM/human experts. The response should be factually correct, precise, and free of errors.
	Comprehensiveness	Completeness of response provided by the LLM/human experts. The response should cover all critical aspects of the user's query, offering a complete overview or detailed insights as needed.
Understanding and reasoning	Understanding	Ability of the LLM/human experts to interpret the user's query correctly. The response should mimic a grasp of meaning, context, and nuances.
	Reasoning	Capability of the LLM/human experts to apply logical processing to generate the response.
Expression style and persona	Clarity	Quality of the response is clear, understandable, and straightforward, making it easy for the user to comprehend the provided response.
Safety and harm	Harm	Quality of response leading to negative outcomes, such as spreading misinformation, reinforcing stereotypes, or otherwise adversely affecting users.
Trust and confidence	Trust	Confidence in the LLM/human experts that it/they will provide accurate, fair, and safe responses. In addition, there is transparency regarding the LLM's/human experts’ capabilities and limitations.

Abbreviation: LLM, large language model.

The clinical application query answers were evaluated based on how well the summary informed the application to a clinical scenario. Each query was scored on a 3‐point Likert scale with the following definitions: (0) incorrect or misaligned with consensus, (1) partially aligned, missing key factors and (2) fully consistent with the consensus and rationale provided.

The misinterpretation resistance query answers were evaluated based on the ability to reject the misleading question. Each query was scored on a 3‐point Likert scale with the following definitions: (0) incorrectly confirms a false statement, (1) provides a hedged or vague correction and (2) rejects the false premise with consensus‐aligned correction.

For both the clinical application and misinterpretation resistance query answers, the three query scores assigned by each reviewer were summarised as a median. The reviewer‐specific median scores were subsequently aggregated by calculating the overall median, which was used for comparison LLM prompting arms and human experts.

### Statistical analysis

For each consensus part, reviewer‐assigned scores were summarized as median, minimum, and maximum values. Differences in median scores between conditions were assessed using the Friedman test for related samples, with each summary QUEST category or clinical scenario answer serving as the unit of analysis. When a significant overall difference was detected, post‐hoc pairwise comparisons were performed using Wilcoxon signed‐rank tests with Holm correction for multiple comparisons. Mean scores and standard deviations are presented in the Appendix S1 for descriptive purposes.

Inter‐rater agreement analyses were conducted separately for each QUEST category by pooling ratings across all consensus parts. Agreement was also assessed separately for the clinical application and misinterpretation resistance items. Inter‐rater agreement was quantified using Gwet's AC2 coefficient with quadratic weights and reported with corresponding 95% confidence intervals (CIs). Agreement levels were interpreted as follows: poor (<0.00), slight (0.00–0.20), fair (0.21–0.40) moderate (0.41–0.60) substantial (0.61–0.80) and almost perfect (0.81–1.00) [10]. All statistical analyses were performed with R software (version 4.5.2; R Foundation for Statistical Computing).

## RESULTS

### Evaluation tasks

#### Evaluation of ChatGPT

For consensus part 1, no significant differences were observed between ChatGPT single‐pass, ChatGPT sequential COT, and human expert outputs across any outcome (Table 2). Descriptively, both ChatGPT conditions received higher numerical median scores than human experts for quality of information, trust and confidence, and misinterpretation resistance queries, whereas ratings were not different across conditions for the remaining outcomes. Mean scores are presented in Supporting Information S1: Appendix Table 1.

**Table 2:** Comparison of outputs from ChatGPT single, and sequential COT prompt and human experts.

Outcome	ChatGPT single‐pass	ChatGPT sequential COT	Human experts	*p*‐value
Consensus part 1				
Quality of information	5 (3–5)	5 (4–5)	4 (3–5)	0.67
Understanding and reasoning	5 (3–5)	5 (4–5)	5 (5–5)	0.37
Expression style and persona	5 (3–5)	5 (4–5)	5 (5–5)	0.37
Safety and harm	5 (4–5)	5 (5–5)	5 (5–5)	0.37
Trust and confidence	5 (3–5)	5 (3–5)	4 (4–5)	0.72
Clinical application queries	2 (1–2)	2 (1–2)	1 (1–2)	0.37
Misinterpretation resistance queries	2 (2–2)	2 (1–2)	1 (1–1)	0.10
Consensus part 2				
Quality of information	3 (3–4)	5 (3–5)	4 (4–5)	0.27
Understanding and reasoning	5 (4–5)	5 (3–5)	5 (5–5)	0.37
Expression style and persona	5 (3–5)	5 (3–5)	5 (5–5)	0.37
Safety and harm	5 (5–5)	5 (4–5)	5 (5–5)	0.37
Trust and confidence	3 (3–4)	5 (3–5)	4 (4–4)	0.27
Clinical application queries	2 (1–2)	2 (2–2)	2 (1–2)	0.61
Misinterpretation resistance queries	2 (2–2)	2 (2–2)	2 (2–2)	[a]

*Note*: All outcomes were presented with median (minimum–maximum).

Abbreviation: COT, chain‐of‐thought.

^a^ All raters rated 2 for all questions.

For consensus part 2, no statistically significant differences were identified between conditions. Descriptively, ChatGPT sequential COT received higher numerical median scores than ChatGPT single‐pass for quality of information and trust and confidence, whereas ratings were not different across conditions for the remaining outcomes. All misinterpretation resistance query responses received maximum ratings, which precluded statistical comparison.

#### Evaluation of claude

For consensus part 1, a statistically significant difference was observed for misinterpretation resistance queries (*p* = 0.0498), whereas no significant differences were identified for the remaining outcomes (Table 3). Descriptively, ratings varied across conditions, where human experts generally received not different or higher numerical ratings for the QUEST categories compared to both Claude conditions, while both Claude conditions received higher numerical median ratings for misinterpretation resistance compared to human experts. However, post‐hoc pairwise comparisons for misinterpretation resistance were not statistically significant. Mean scores are presented in Supporting Information S1: Appendix Table 2.

**Table 3:** Comparison of outputs from Claude single‐pass, and sequential COT prompt and human experts.

Outcome	Claude single‐pass	Claude sequential COT	Human experts	*p*‐value
Consensus part 1				
Quality of information	4 (3–4)	3 (3–4)	4 (3–5)	0.37
Understanding and reasoning	4 (4–4)	3 (2–4)	5 (5–5)	0.06
Expression style and persona	5 (4–5)	5 (2–5)	5 (5–5)	0.37
Safety and harm	4 (4–4)	4 (2–4)	5 (5–5)	0.06
Trust and confidence	4 (3–4)	3 (3–4)	4 (4–5)	0.22
Clinical application queries	2 (1–2)	1 (0–1)	1 (1–2)	0.10
Misinterpretation resistance queries	2 (2–2)^a^	2 (2–2)^a^	1 (1–1)^a^	0.0498^a^
Consensus part 2				
Quality of information	4 (4–5)	5 (3–5)	4 (4–5)	0.72
Understanding and reasoning	5 (4–5)	4 (3–5)	5 (5–5)	0.37
Expression style and persona	5 (4–5)	4 (2–4)	5 (5–5)	0.10
Safety and harm	5 (5–5)	5 (5–5)	5 (5–5)	[b]
Trust and confidence	4 (3–4)	5 (3–5)	4 (4–4)	0.67
Clinical application queries	2 (2–2)	2 (2–2)	2 (1–2)	0.37
Misinterpretation resistance queries	2 (2–2)	2 (2–2)	2 (2–2)	[c]

*Note*: All outcomes were presented with median (minimum–maximum).

Abbreviation: COT, chain‐of‐thought.

^a^ Pairwise comparisons were non‐significant between Claude single‐pass and Claude sequential COT compared to human experts (*p* = 0.083).

^b^ All raters rated 5 for all questions.

^c^ All raters rated 2 for all questions.

For consensus part 2, no statistically significant differences were identified between conditions. Descriptively, ratings were generally not different across conditions. The QUEST category safety and harm and all misinterpretation resistance query responses received maximum ratings, which precluded statistical comparison.

#### Evaluation of Gemini

For consensus part 1, a statistically significant difference was observed for misinterpretation resistance queries (*p* = 0.0498), whereas no significant differences were identified for the remaining outcomes (Table 4). Descriptively, ratings were generally not different across conditions, with human experts receiving not different or higher numerical ratings for most QUEST categories compared to both Gemini conditions, while both Gemini approaches received higher numerical median ratings for misinterpretation resistance compared to human experts. However, post‐hoc pairwise comparisons for misinterpretation resistance were not statistically significant. Mean scores are presented in Supporting Information S1: Appendix Table 3.

**Table 4:** Comparison of outputs from Gemini single‐pass, and sequential COT prompt and human experts.

Outcome	Gemini single‐pass	Gemini sequential COT	Human experts	*p*‐value
Consensus part 1				
Quality of information	4 (4–5)	4 (4–4)	4 (3–5)	0.61
Understanding and reasoning	4 (3–4)	4 (4–4)	5 (5–5)	0.06
Expression style and persona	5 (5–5)	5 (4–5)	5 (5–5)	0.37
Safety and harm	4 (4–4)	4 (3–5)	5 (5–5)	0.15
Trust and confidence	3 (3–4)	4 (4–4)	4 (4–5)	0.16
Clinical application queries	1 (1–1)	1 (1–2)	1 (1–2)	0.61
Misinterpretation resistance queries	2 (2–2)^a^	2 (2–2)^a^	1 (1–1)^a^	0.0498^a^
Consensus part 2				
Quality of information	3 (3–4)	5 (4–5)	4 (4–5)	0.18
Understanding and reasoning	4 (3–4)	5 (3–5)	5 (5–5)	0.20
Expression style and persona	5 (4–5)	5 (3–5)	5 (5–5)	0.37
Safety and harm	5 (4–5)	5 (5–5)	5 (5–5)	0.37
Trust and confidence	3 (3–4)	5 (3–5)	4 (4–4)	0.53
Clinical application queries	2 (1–2)	2 (1–2)	2 (1–2)	>0.99
Misinterpretation resistance queries	2 (2–2)	2 (2–2)	2 (2–2)	[b]

*Note*: All outcomes were presented with median (minimum–maximum).

Abbreviation: COT, chain‐of‐thought.

^a^ Pairwise comparisons were non‐significant between Claude single‐pass and Claude sequential COT compared to human experts (*p* = 0.083).

^b^ All raters rated 2 for all questions.

For consensus part 2, no statistically significant differences were identified between conditions. Descriptively, Gemini sequential COT received higher numerical median ratings than single‐pass prompting and human experts for quality of information and trust and confidence, while ratings were generally not different across conditions for the remaining outcomes. All misinterpretation resistance query responses received maximum ratings, which precluded statistical comparison.

#### Inter‐rater reliability

The inter‐rater agreement was deemed almost perfect for the QUEST categories of quality of information, understanding and reasoning, expression style and persona, safety and harm, as well as for the clinical application, and misinterpretation resistance queries, whereas the QUEST category trust and confidence was deemed substantial (Table 5).

**Table 5:** Inter‐rater agreement between reviewers for each QUEST category and the clinical application and misinterpretation resistance queries.

Category	Gwet's AC2 (95% Confidence interval)	Agreement level
Quality of information	0.81 (0.72; 0.91)	Almost perfect
Understanding and reasoning	0.83 (0.72; 0.94)	Almost perfect
Expression style and persona	0.81 (0.67; 0.96)	Almost perfect
Safety and harm	0.95 (0.88; 1.00)	Almost perfect
Trust and confidence	0.79 (0.67; 0.92)	Substantial
Clinical application queries	0.96 (0.89; 1.00)	Almost perfect
Misinterpretation resistance queries	0.99 (0.99; 1.00)	Almost perfect

*Note*: All outcomes were presented with Gwet's AC2 estimate with 95% confidence interval and agreement level.

## DISCUSSION

The main findings of this study were that outputs generated by ChatGPT, Claude, Gemini, and human experts received high ratings across most QUEST evaluation categories, with no statistically significant differences observed between conditions and no indication of superior performance for any condition. Human expert and LLM‐generated summaries generally received not significantly different ratings, although sequential COT prompting frequently received higher numerical median ratings than single‐pass prompting, particularly for quality of information and trust and confidence; the observed advantages of sequential COT prompting were primarily numerical rather than statistically significant.

Across the evaluated categories, the relative performance of the generation strategies differed between LLM families. For ChatGPT, the sequential COT approach generally received the highest numerical ratings, followed by the single‐pass approach, and human expert output received slightly lower numerical ratings overall. For Claude, single‐pass outputs and human expert outputs did not significantly differently and generally received higher numerical ratings than the sequential COT approach. For Gemini, the sequential COT approach generally received the highest numerical ratings, followed by human expert outputs, whereas the single‐pass approach tended to receive the lowest numerical ratings. The high level of inter‐rater agreement supports the robustness of the human evaluation framework used in this study. Agreement was almost perfect across most QUEST categories and substantial for trust and confidence, which indicates that reviewer assessments were generally consistent. Consequently, the observed rating patterns between LLM‐generated and human expert‐generated summaries are unlikely to be attributable to reviewer variability alone.

### LLMs versus human experts

The generally high ratings observed for LLM‐generated summaries are consistent with previous research that evaluated both medical information synthesis and clinical reasoning. Van Veen et al. [25] reported that GPT‐4‐generated clinical summaries were frequently preferred over summaries produced by medical experts when assessed by blinded physician reviewers, particularly for completeness, correctness, and conciseness. McDuff et al. [11] reported improved diagnostic accuracy for physicians who utilised an LLM as an adjunct tool compared to physicians with or without adjunct traditional searching. Conversely, in a randomised controlled trial, Goh et al. [8] reported that physician diagnostic accuracy did not improve with adjunct use of an LLM compared to conventional methods, although the LLM outperformed the physicians when used in isolation. Furthermore, Brodeur et al. [3] reported that a reasoning‐optimised LLM outperformed physician baselines across multiple diagnostic and management reasoning benchmarks, which included evaluations using real‐world emergency department cases. Although the present study evaluated summarisation of a meniscus treatment consensus rather than diagnostic decision‐making, the consistently high ratings for understanding and reasoning observed across LLM‐generated summaries were broadly aligned with the results from Brodeur et al. [3]. Collectively, the available data suggest that LLMs are increasingly capable of both synthesizing complex medical knowledge and presenting that information in a clinically meaningful manner, especially compared to human recommendations.

### Sequential chain‐of‐thought versus single‐pass prompting

A notable observation was that sequential COT prompting received higher numerical ratings than single‐pass prompting for ChatGPT and Gemini, particularly in categories related to quality of information and trust and confidence. In contrast, higher numerical ratings with sequential COT prompting were not consistently observed for Claude, where single‐pass outputs and human expert outputs generally received higher numerical ratings than the sequential COT configuration. These findings suggest that the effectiveness of sequential COT prompting may be model‐dependent rather than universally beneficial.

The observed advantages of sequential COT prompting for selected models are consistent with the theoretical rationale underlying agent‐based architectures [14]. Rather than generation of a response through a single inference step, sequential COT systems typically distribute subtasks across specialized agents that iteratively generate, critique, and refine outputs before production of a final response. Sequential COT architecture may improve reasoning performance, factual accuracy, and decision‐making quality by enabling multiple independent perspectives to be incorporated before a final answer is generated [7, 13, 14]. However, the inconsistent findings observed for Claude suggest that increased architectural complexity alone does not guarantee improved performance. Previous data demonstrates substantial performance variation across contemporary AI systems, with frontier foundation models that outperformed specialized clinical AI tools across medical knowledge, clinician‐alignment, and real‐world clinical query benchmarks [27]. Together with the present findings, the benefits of advanced prompting strategies may depend not only on the prompting framework itself but also on the underlying model architecture.

### Clinical perspective

From a practical perspective, the findings suggest that contemporary LLMs can effectively synthesize complex sports medicine consensuses into concise summaries while maintaining high ratings across several clinically relevant domains. The consistently favourable ratings across several QUEST categories indicate that LLM‐generated summaries were generally perceived as understandable, logically structured, and favourable with regard to safety and harm. Although greater variability was observed for quality of information and trust and confidence, median ratings remained high across most conditions, which suggests that key recommendations from the original consensuses were largely preserved.

The results from this study highlight the potential role of LLMs as knowledge translation tools in clinical practice. Clinicians are frequently required to interpret lengthy consensuses and guidelines under time constraints. Summaries generated by LLMs and the ability to pose follow‐up clinical questions may have the potential to facilitate access to key recommendations and provide context‐specific guidance. However, clinician efficiency and decision quality were not evaluated in the present study and should be considered hypotheses for future research. At this point, LLM‐generated outputs should be viewed as adjuncts to, rather than replacements for, clinical expertise and reasoning. Final clinical decisions should continue to integrate individual patient characteristics, clinician judgement, and consultation of the original source documents when necessary. The present findings therefore support the potential clinical usefulness of LLMs as knowledge translation tools, while further studies are needed to determine the effects of LLM use on clinical efficiency and decision‐making.

### Limitations

The reproducibility of the generated output was not assessed. As LLM responses may vary across repeated interactions, future studies should evaluate the consistency of summary generation and clinical recommendations across multiple runs. Second, the evaluated LLM versions represent a rapidly evolving technology and may no longer reflect the current state of the art. However, given the generally high ratings observed across evaluation categories, newer model versions would be expected to maintain or improve upon the performance reported in the present study. Third, the number of generated summaries, clinical scenario responses, and participating human experts was limited, which reduced statistical power and created uncertainty with regard to the most appropriate independent unit of analysis and representativeness of human‐written outputs. Furthermore, the human experts and reviewers participated in the development of the evaluated consensus, which may have influenced assessments and outputs. Therefore, non‐significantly different findings should not be interpreted as evidence of equivalence between conditions. Fourth, the clinical scenario responses were generated from the preceding summaries. Consequently, the study design could not distinguish errors that arose during summarisation from errors that occurred during the application of summarised information to clinical questions. Fifth, although the evaluation framework was informed by the QUEST methodology, the three‐ to five‐point rating scales used for summary evaluation and query assessment have not been formally validated. Furthermore, the overtly misleading clinical scenarios and relatively coarse scoring criteria may have introduced ceiling effects and may not reflect resistance to more subtle, clinically plausible, or complex forms of misinterpretation. Finally, only one sports medicine consensus was evaluated, which addressed a single clinical topic and originated from the one consensus initiative with similar structure, terminology, and authorship. Therefore, the findings may not generalise to other clinical topics, guideline formats, and consensus development processes.

## CONCLUSION

No statistically significant differences in reviewer ratings were observed between ChatGPT, Claude, Gemini and human expert output, with generally high ratings for summaries and responses to clinical scenarios. Large language models may therefore represent a potential adjunct for summarisation and interpretation of sports medicine consensus recommendations to inform rehabilitation decision‐making.

## AUTHOR CONTRIBUTIONS

Jakob Lindskog contributed to the design project, drafted the initial version of the manuscript, performed analysis of data, has approved the final work for publication, and has agreed to be accountable for all aspects of the work. Felix Öttl and Yinan Yu contributed to the design project, contributed majorly during the analysis and interpretation of data, made important contributions during the drafting of the work, approved the final work, and agreed to be accountable for all aspects of the work. Mark V. Paterno, Airelle Giordano and Alexandre Rambaud were part of the human expert panel, made meaningful contributions during the final stages of manuscript drafting, approved the final work, and agreed to be accountable for all aspects of the work. Robert Prill and Aleksandra Królikowska were part of the reviewer panel, made meaningful contributions during the final stages of manuscript drafting, approved the final work, and agreed to be accountable for all aspects of the work. Kristian Samuelsson contributed during the interpretation of data, made meaningful contributions during the final stages of manuscript drafting, approved the final work, and agreed to be accountable for all aspects of the work. Eric Hamrin Senorski was part of the reviewer panel, contributed majorly during the drafting of the manuscript, analysis and interpretation of data, is responsible for the design concept, approved the final work, and agreed to be accountable for all aspects of the work.

## FUNDING

The authors have no funding to report.

## CONFLICT OF INTEREST STATEMENT

Author Kristian Samuelsson reports a relationship with Getinge AB that includes: board membership. The authors declare no conflicts of interest.

## ETHICS STATEMENT

The authors have nothing to report.

## Supporting information

Appendix AI Prompting Meniscus Consensus.

## Data Availability

The data that support the findings of this study are available from the corresponding author upon reasonable request.
