# Supplementary material for: No significant differences between large language model prompting strategies and human experts in summarisation of ‘the formal EU–US meniscus rehabilitation consensus: An ESSKA‐AOSSM‐AASPT Initiative’
Source: J Exp Orthop. 2026 Oct 28;13(4):e70964. doi: 10.1002/jeo2.70964 (PMC13715498; doi:10.1002/jeo2.70964)
Supplement: Supplementary file 1 [file JEO2-13-e70964-s001.docx]

| Appendix table 1: Mean and standard deviation of outputs from ChatGPT single-pass, and sequential COT prompt and human experts. | | | |
| --- | --- | --- | --- |
| Outcome | ChatGPT single-pass | ChatGPT sequential COT | Human experts |
| ESSKA consensus statement part 1 |  |  |  |
| Quality of information | 4.3 ± 1.2 | 4.7 ± 0.6 | 4.0 ± 1.0 |
| Understanding and reasoning | 4.3 ± 1.0 | 4.7 ± 0.6 | 5.0 ± 0.0 |
| Expression style and persona | 4.3 ± 1.2 | 4.7 ± 0.6 | 5.0 ± 0.0 |
| Safety and harm | 4.7 ± 0.6 | 5.0 ± 0.0 | 5.0 ± 0.0 |
| Trust and confidence | 4.3 ± 1.2 | 4.3 ± 1.2 | 4.3 ± 0.6 |
| Clinical application queries | 1.7 ± 0.6 | 1.7 ± 0.3 | 1.2 ± 0.5 |
| Misinterpretation resistance queries | 2.0 ± 0.0 | 1.8 ± 0.4 | 1.3 ± 0.0 |
| ESSKA consensus statement part 2 |  |  |  |
| Quality of information | 3.3 ± 0.6 | 4.3 ± 1.2 | 4.3 ± 0.6 |
| Understanding and reasoning | 4.7 ± 0.6 | 4.3 ± 1.2 | 5.0 ± 0.0 |
| Expression style and persona | 4.3 ± 1.2 | 4.3 ± 1.2 | 5.0 ± 0.0 |
| Safety and harm | 5.0 ± 0.0 | 4.7 ± 0.6 | 5.0 ± 0.0 |
| Trust and confidence | 3.3 ± 0.6 | 4.3 ± 1.2 | 4.0 ± 0.0 |
| Clinical application queries | 1.6 ± 0.2 | 1.8 ± 0.2 | 1.4 ± 0.4 |
| Misinterpretation resistance queries | 2.0 ± 0.0 | 2.0 ± 0.0 | 2.0 ± 0.0 |
| COT = Chain-of-thought |  |  |  |
|  | | | |

| Appendix table 2: Mean and standard deviation of outputs from Claude single-pass, and sequential COT prompt and human experts. | | | |
| --- | --- | --- | --- |
| Outcome | Claude single-pass | Claude sequential COT | Human experts |
| ESSKA consensus statement part 1 |  |  |  |
| Quality of information | 3.7 ± 0.6 | 3.3 ± 0.6 | 4.0 ± 1.0 |
| Understanding and reasoning | 4.0 ± 0.0 | 3.0 ± 1.0 | 5.0 ± 0.0 |
| Expression style and persona | 4.7 ± 0.6 | 4.0 ± 1.7 | 5.0 ± 0.0 |
| Safety and harm | 4.0 ± 0.0 | 3.3 ± 1.2 | 5.0 ± 0.0 |
| Trust and confidence | 3.7 ± 0.6 | 3.3 ± 0.6 | 4.3 ± 0.6 |
| Clinical application queries | 1.6 ± 0.5 | 0.9 ± 0.5 | 1.2 ± 0.5 |
| Misinterpretation resistance queries | 2.0 ± 0.0 | 1.8 ± 0.2 | 1.3 ± 0.0 |
| ESSKA consensus statement part 2 |  |  |  |
| Quality of information | 4.3 ± 0.6 | 4.3 ± 1.2 | 4.3 ± 0.6 |
| Understanding and reasoning | 4.7 ± 0.6 | 4.0 ± 1.0 | 5.0 ± 0.0 |
| Expression style and persona | 4.7 ± 0.6 | 3.3 ± 1.2 | 5.0 ± 0.0 |
| Safety and harm | 5.0 ± 0.0 | 5.0 ± 0.0 | 5.0 ± 0.0 |
| Trust and confidence | 3.7 ± 0.6 | 4.3 ± 1.2 | 4.0 ± 0.0 |
| Clinical application queries | 1.9 ± 0.2 | 1.9 ± 0.2 | 1.4 ± 0.4 |
| Misinterpretation resistance queries | 2.0 ± 0.0 | 2.0 ± 0.0 | 2.0 ± 0.0 |
| COT = Chain-of-thought |  |  |  |

| Appendix table 3: Mean and standard deviation of outputs from Gemini single-pass, and sequential COT prompt and human experts. | | | |
| --- | --- | --- | --- |
| Outcome | Gemini single-pass | Gemini sequential COT | Human experts |
| Consensus part 1 |  |  |  |
| Quality of information | 4.3 ± 0.6 | 4.0 ± 0.0 | 4.0 ± 1.0 |
| Understanding and reasoning | 3.7 ± 0.6 | 4.0 ± 0.0 | 5.0 ± 0.0 |
| Expression style and persona | 5.0 ± 0.0 | 4.7 ± 0.6 | 5.0 ± 0.0 |
| Safety and harm | 4.0 ± 0.0 | 4.0 ± 1.0 | 5.0 ± 0.0 |
| Trust and confidence | 3.3 ± 0.6 | 4.0 ± 0.0 | 4.3 ± 0.6 |
| Clinical application queries | 0.9 ± 0.4 | 1.2 ± 0.4 | 1.2 ± 0.5 |
| Misinterpretation resistance queries | 1.9 ± 0.2 | 2.0 ± 0.0 | 1.3 ± 0.0 |
| Consensus part 2 |  |  |  |
| Quality of information | 3.3 ± 0.6 | 4.7 ± 0.6 | 4.3 ± 0.6 |
| Understanding and reasoning | 3.7 ± 0.6 | 4.3 ± 1.2 | 5.0 ± 0.0 |
| Expression style and persona | 4.7 ± 0.6 | 4.3 ± 1.2 | 5.0 ± 0.0 |
| Safety and harm | 4.7 ± 0.6 | 5.0 ± 0.0 | 5.0 ± 0.0 |
| Trust and confidence | 3.3 ± 0.6 | 4.3 ± 1.2 | 4.0 ± 0.0 |
| Clinical application queries | 1.7 ± 0.3 | 1.6 ± 0.5 | 1.4 ± 0.4 |
| Misinterpretation resistance queries | 2.0 ± 0.0 | 2.0 ± 0.0 | 2.0 ± 0.0 |
| COT = Chain-of-thought |  |  |  |
|  | | | |
